# Hepatitis B core antibody positivity is not associated with risk of transaminase elevation following switch to dual antiretroviral therapy

**DOI:** 10.1097/QAD.0000000000004227

**Published:** 2025-07-01

**Authors:** Luca Mezzadri, Alessandro Guido Soria, Alice Ranzani, Sergio Malandrin, Francesca Sabbatini, Silvia Limonta, Anna Cappelletti, Elisa Colella, Nicola Squillace, Ilaria Chiara Giuseppina Caramma, Bianca Monti, Alban Rugova, Annalisa Cavallero, Paolo Bonfanti, Giuseppe Lapadula

**Affiliations:** a School of Medicine and Surgery, University of Milano-Bicocca, Milan; b Infectious Diseases Unit; c Microbiology Unit, Fondazione IRCCS San Gerardo Dei Tintori, Monza, Italy.

**Keywords:** anti-HBc positivity, HIV, liver function test increases, occult hepatitis B infection, two-drug regimens

## Abstract

**Objective:**

To evaluate whether hepatitis B core antibodies, indicative of possible occult hepatitis B virus (HBV) infection (pOBI), are associated with an increased risk of transaminase elevation in people with HIV (PWH) switching to two-drug regimens (2DR).

**Design:**

Cohort study.

**Methods:**

We included PWH who switched to 2DR since 2018, if they discontinued at least one anti-HBV drug and were HBsAg-negative at baseline. Two cohorts were analyzed: cohort 1 discontinued tenofovir (TFV) but continued lamivudine (3TC), Cohort 2 switched to regimens without HBV-active drugs. Survival analysis, including Cox regression adjusting for potential confounders, was conducted to compare time to grade ≥1 transaminase increase in those with and without pOBI.

**Results:**

Among 167 patients in cohort 1 (35.2% with pOBI), the rate of transaminitis was 4.59 vs. 7.47 per 100 person-years for those with and without pOBI [incidence rate ratio (IRR) 0.61; 95% confidence interval (CI) 0.17–1.83; *P* = 0.259]. Cox multivariable analysis found no significant association between pOBI and transaminitis (hazard ratio 0.56; 95% CI 0.2–1.5; *P* = 0.266), with adjusted models confirming these results. Among 118 individuals in cohort 2 (33.9% with pOBI), transaminitis rates were 8.04 vs. 8.68 per 100 person-years for those with and without pOBI (IRR 0.93; 95% CI 0.19–3.91; *P* = 0.763). Cox regression showed no significant association between pOBI and transaminitis (hazard ratio 1.18; 95% CI 0.4-3.6; *P* = 0.769), with consistent findings in adjusted models. No HBV reactivation occurred in either cohort.

**Conclusion:**

pOBI was not associated with risk of transaminase elevation in PWH switching to dual therapies lacking anti-HBV agents.

## Introduction

According to WHO estimates, approximately 39.9 million individuals worldwide were living with HIV by the end of 2023 [1]. A significant proportion of this population is co-infected with the hepatitis B virus (HBV), since transmission routes are shared.

Following exposure to HBV, the persistence of covalently closed circular DNA in the liver in patients testing negative for HBV surface antigen (HBsAg) defines the status of occult HBV infection (OBI) and represents a potential risk for future viral reactivation. In clinical practice, HBV core antibody (HBcAb) is commonly used as a surrogate marker to diagnose potential OBI (pOBI) [2]. The prevalence of HBcAb positivity among people with HIV (PWH) is not precisely defined, but it is estimated to range between 10 and 45% in different geographical contexts [3–6].

While guidelines recommend including antiretroviral medications effective against HBV in the treatment regimens for PWH who are HBsAg-positive, the management of those with pOBI remains less clearly defined [7]. Concerns have been raised regarding potential HBV reactivations among PWH receiving dual therapies without anti-HBV activity, as HBV reactivations following the cessation of HBV-active antiretrovirals have been noted. However, to date, confirmed cases of pOBI reactivation after stopping anti-HBV therapy have been reported primarily in PWH with severe CD4^+^ T-cell depletion or drug-induced immunosuppression [8–11].

While such cases highlight potential risks, compelling evidence for HBV reactivation risk in patients not meeting these specific criteria is limited. Nonetheless, sporadic cases of HBV reactivation after switching to regimens without HBV-active agents have been documented, even in individuals without obvious risk factors, such as low CD4^+^ counts or immunosuppression. For instance, a case of clinically significant reactivation was reported in a patient with isolated anti-HBc positivity with high CD4^+^ counts and no evidence of reinfection risks. This event, characterized by HBsAg reversion, increased HBV DNA levels, and mild transaminase elevation, occurred following the transition to a long-acting regimen, raising concerns that subclinical or latent HBV replication may occasionally escape immune control, even in the absence of clear predisposing factors [12]. The transition to regimens without HBV-active drugs is becoming increasingly common with the approval of long-acting regimens. Some experts argue that this practice should be avoided in patients with pOBI, potentially restricting access to beneficial treatment options. As a reflection, ongoing trials on dual therapies without HBV-activity often exclude HBcAb-positive patients from participation [13]. By converse, current European guidelines mandate the inclusion of active HBV drugs in antiretroviral regimens for PWH with pOBI only in cases of severe immunodepression, while, in other situations, careful monitoring of HBV DNA levels is advised [7]. Although this approach is less stringent than avoiding treatment without HBV-active drugs altogether, it is important to acknowledge that even this recommendation is not strongly supported by evidence and could potentially be unnecessary. In the end, the actual risk of clinically significant HBV reactivation in individuals switching to dual regimens remains poorly quantified but likely very low. This raises the possibility that the perceived risks may be overestimated, and the benefits of switching to simplified or long-acting regimens for individuals with pOBI could outweigh the minimal potential for reactivation.

HBV reactivation is primarily considered a virologic event. However, while a rise in plasma HBV DNA is often used as a diagnostic marker, its measurement is not routinely performed in clinical practice. As a result, transaminase levels may serve as a practical and reliable indicator for detecting hepatitis flares [14]. To address the current gap in knowledge, we aimed at comparing the risk of transaminase elevations in PWH with and without pOBI who transition to dual antiretroviral therapies lacking anti-HBV agents. By directly comparing these groups, we aim to quantify the actual relative risk of liver inflammation in the context of pOBI and estimate whether pOBI confers a clinically meaningful risk of hepatic events.

## Methods

### Study design

We conducted a post hoc analysis of the data of patients from a prospectively maintained cohort, the HSG (Hospital San Gerardo) Cohort Study. The HSG Cohort Study is a prospective observational cohort of PWH receiving care at Fondazione IRCCS San Gerardo dei Tintori, a tertiary referral academic hospital located in Italy. Upon enrollment, participants provide written informed consent, their clinical data are recorded at baseline and clinical visits, treatment data, laboratory values, and plasma leftover samples are prospectively collected using an electronic clinical record system routinely used for patient care, which also functions as a structured database for research purposes. The study was approved by the local institutional review board (Comitato Etico Brianza) under protocol number 245, on 22 June 2017.

For the purpose of the present analysis, patients were eligible if they had introduced for the first time a dual regimen between January 2018 and October 2023, discontinuing at least one drug with anti-HBV activity among tenofovir disoproxil-fumarate or alafenamide-fumarate (TxF) and lamivudine or emtricitabine (xTC). Additionally, patients were required to be negative for hepatitis B surface antigen (HBsAg) at baseline and to have HBcAb and transaminase levels measured before the switch. Two separate cohorts were studied: cohort 1 comprised patients who discontinued tenofovir (TxF) but continued xTC; cohort 2 consisted of patients who switched to a regimen that excluded both TxF and xTC.

### Outcome measures

The primary outcome measure was the time to grade ≥1 liver function test increase (LFTI), defined as alanine aminotransferase (ALT) or aspartate aminotransferase (AST) level at least 1.25 times the upper limit of normal, in accordance with the Division of AIDS Table for Grading the Severity of Adult and Pediatric Adverse Events [15]. Specifically, the upper limits of normal used in our analyses were 41 IU/l for ALT and 40 IU/l for AST in men, and 33 IU/l for ALT and 32 IU/l for AST in women.

Secondary outcomes measures included time to grade ≥3 LFTI (i.e. more than five times the upper limit of normal), transaminase evolution over time after the switch and the rate of transaminase elevations during follow-up. Transaminase levels were typically measured every 4–6 months, in accordance with routine clinical practice. For the purpose of analysis, wherever necessary, values were grouped into 6-month intervals (±90 days) to account for variability in follow-up timing and ensure comparability across participants.

### Statistical analysis

All analyses were conducted separately for cohort 1 and cohort 2.

In the primary analysis, we used Kaplan–Meier curves to visualize the time to grade ≥1 LFTI and the log-rank test along with Cox regression models to evaluate the impact of both categorical and continuous covariates on time to LFTI. The main exposure of interest was the presence of pOBI, as indicated by HBcAb serum reactivity. Additional covariates included sex, age, ethnicity, risk factor for HIV acquisition, hepatitis C virus antibody (HCVAb) and HBV surface antibodies (HBsAb) serostatus, alcohol abuse, diabetes, BMI, calendar year of therapy switch, baseline CD4^+^ count, baseline levels of AST and ALT, and type of antiretroviral therapy. Alcohol use was self-reported during structured interviews, and alcohol abuse was defined as either reporting binge drinking (at least five drinks in 24 h) or consuming more than 10 drinks per week. Follow-up began at the time of the switch and continued until LFTI occurred or until the last recorded AST/ALT measurement, whichever came first. Patients were right-censored if they modified their regimen by reintroducing HBV-active drugs.

Transaminase trajectories were compared using mixed-effects linear regression models with random intercepts and slopes, accounting for both time and core antibody status. Generalized Estimating Equation (GEE) models with exchangeable correlation structure were used to compare the rates of grade ≥1 liver LFTI, based on repeated measures of transaminases.

Missing data for HCVAb, HBsAb, alcohol use, BMI, and HIV risk factor were handled using the Multiple Imputation by Chained Equations (MICE) approach, generating 20 imputed datasets.

All statistical analyses were conducted using Stata version 18 (StataCorp, College Station, Texas, USA). A two-tailed *P* value below 0.05 indicated conventional statistical significance.

## Results

### Patient characteristics

A total of 285 patients who had switched to a dual therapy discontinuing HBV-active drugs were identified. Among them, 167 switched to a regimen containing xTC (cohort 1), while 118 switched to a regimen excluding both TxF and xTC (cohort 2). The median number of transaminase measurements after treatment switch was 5 [interquartile range (IQR) 4–6] for patients in cohort 1, and 4 (IQR: 3–6) for patients in cohort 2. Table 1 shows the characteristics of the patients included in each cohort, grouped by pOBI status. Most patients were male, with 82% of males in cohort 1 and 70% in cohort 2, and born in Italy (83% in cohort 1 and 84% in cohort 2). In both cohorts, those with pOBI were significantly older than those without it (mean age 54.6 years vs. 45.6 in cohort 1 and 56 years vs 48.7 in cohort 2, respectively). Additionally, patients with pOBI, compared to those without it, were significantly more likely to have a history of intravenous drug use and to test positive for HCVAb. However, none had detectable HCV RNA in plasma, indicating the absence of active hepatitis C virus infection.

**Table 1:** Patients’ characteristics.

	Cohort 1 (*N* = 167): dual regimens with 3TC			Cohort 2 (N = 118): dual regimens without anti-HBV drugs		
Parameter	No pOBI (108)	pOBI (59)	*P*	No pOBI (78)	pOBI (40)	*P*
Male sex (*n*, %)	85 (78.7)	50 (84.8)	0.343	53 (68)	29 (72.5)	0.611
Age (mean; SD)	45.6 (11.1)	54.6 (11.9)	<0.001	48.7 (12.4)	56 (8.5)	0.001
Born in Italy (*n*, %)	96 (88.9)	46 (78)	0.059	64 (82.1)	34 (85)	0.686
Risk factor for HIV acquisition (*n*, %)			0.019			0.005
Heterosexual	53 (53.5)	22 (44)		40 (62.5)	15 (42.8)	
MSM	44 (44.4)	22 (44)		22 (34.4)	10 (28.6)	
Intravenous drug use	1 (1)	6 (12)		2 (3.1)	7 (20)	
Other	1 (1)	0		0	3 (8.6)	
HBsAb positive (*n*, %)	61 (56.5)	43 (74.1)	0.018	35 (46.1)	26 (72.2)	0.006
HCV Ab positive (*n*, %)^a^	10 (9.3)	10 (17.5)	0.037	6 (8)	10 (26.3)	0.008
Alcohol abuse (*n*, %)^$^	13 (14.1)	11 (22.9)	0.349	13 (20.6)	6 (15.8)	0.089
Diabetes (*n*, %)	11 (10.2)	8 (13.6)	0.512	5 (6.4)	6 (15)	0.180
BMI (median; IQR)	24.6 (22.2–27.9)	25.9 (23.5–29.1)	0.099	25 (21.3–27.7)	26.1 (21.5–28.1)	0.611
Calendar year of switch (*n*, %)			0.058			0.185
2018–2019	2 (1.9)	6 (10.2)		6 (7.7)	5 (12.5)	
2020–2021	64 (59.3)	34 (57.6)		23 (29.5)	17 (42.5)	
2022–2023	42 (38.9)	19 (32.2)		49 (62.8)	18 (45)	
CD4^+^ cell count at switch (median cells/μl; IQR)	781 (562–970)	764 (429–938)	0.389	808 (514–1120)	721 (367–1055)	0.264
HIV-RNA at switch (*n*, %)			0.402			0.311
≤50 copies/ml	106 (98.1)	57 (98.3)		65 (85.5)	34 (87.2)	
51–200 copies/ml	1 (0.9)	0 (0)		3 (3.9)	4 (10.3)	
201–1000 copies/ml	1 (0.9)	0 (0)		2 (2.6)	0 (0)	
>1000 copies/ml	0 (0)	1 (1.7)		6 (7.9)	1 (2.6)	
Previous antiretroviral regimen (*n*,%)			0.751			0.985
NRTI + NNRTI	26 (24.1)	10 (16.9)		29 (37.5)	19 (47.5)	
NRTI + bPI	3 (2.8)	5 (8.5)		9 (11.5)	0 (0)	
NRTI + INSTI	78 (72.2)	44 (74.6)		39 (50)	17 (42.5)	
NRTI + INSTI + bPI	1 (0.9)	0		1 (1.3)	4 (10)	
Type of therapy after regimen switch (*n*; %)			N.A.			0.619
3TC+DTG	108 (100)	59 (100)		-	-	
RPV+DTG	–	–		29 (37.2)	19 (47.5)	
RPV+CAB	–	–		38 (48.7)	13 (32.5)	
Other	–	–		11 (14.1)	8 (20)	
Anti-HBV drug discontinuation (*n*, %)			0.078			0.856
TDF ± FTC stop	17 (15.7)	16 (27.1)		7 (9)	4 (10)	
TAF ± FTC stop	91 (84.3)	43 (72.9)		40 (51.3)	21 (52.5)	
Only 3TC stop	NA	NA		31 (39.7)	15 (37.5)	
Follow-up time, years (median; IQR)	1.7 (0.9–2.7)	2.3 (0.9–2.8)		0.7 (0.3–1.5)	1.2 (0.4–2.5)	

a HCV-RNA was undetectable in all patients, indicating previous HCV infection $. 3TC, lamivudine; bPI, boosted protease inhibitor; CAB, cabotegravir; DTG, dolutegravir; FTC, emtricitabine; HBsAb, hepatitis B surface antibody; HCV Ab, hepatitis C virus antibody; INSTI, integrase strand transfer inhibitor; IQR, interquartile range; NNRTI, non-nucleoside reverse transcriptase inhibitor; NRTI, nucleoside reverse transcriptase inhibitor; pOBI, potential occult hepatitis B infection; RPV, rilpivirine; SD, standard deviation; TAF, tenofovir alafenamide fumarate; TDF, tenofovir disoproxil fumarate.

### Risk of transaminase increase after switching to dual regimens

Following the switch to dual regimens, patients were observed for a mean duration of 1.76 years (95% CI 1.59–1.93) in cohort 1 and 1.06 years (95% CI 0.86–1.25) in cohort 2. In cohort 1, 20 patients experienced grade ≥1 LFTI, accounting for an incidence of 6.03 per 100 person-years of follow-up. Similarly, 13 events were observed in cohort 2, corresponding to a rate of 8.36 per 100 person-years of follow-up. No grade ≥3 LFTI were observed in either cohort.

**Fig. 1:**
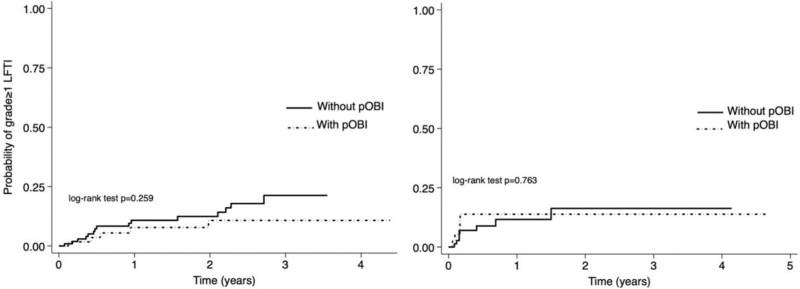
Kaplan–Meier curves showing the probability of experiencing grade ≥1 liver function test increase over time in those switching to lamivudine-containing regimens (left panel) and in those switching to regimens without any HBV drugs (right panel), stratified by potential occult HBV infection.

LFTI, liver function test increase; pOBI, potential occult hepatitis B virus infection.

As shown in Fig. 1 (left panel), among patients included in cohort 1, the incidence of grade ≥1 LFTI was not significantly different between those with or without pOBI [4.59 vs. 7.47 per 100 person-years of follow-up, respectively; incidence rate ratio (IRR) 0.61; 95% CI 0.17–1.83; log-rank test *P* = 0.259). In cohort 2 (Fig. 1, right panel), the incidence of grade ≥1 LFTI was 8.04 per 100 person-years for those with pOBI and 8.68 per 100 person-years for those without pOBI (IRR 0.93; 95% CI 0.19–3.91; log-rank test *P* = 0.763).

Cox regression analyses corroborated these findings, showing no significant association between pOBI and LFTI risk in either cohort 1 (hazard ratio 0.56; 95% CI 0.2–1.5; *P* = 0.266) or cohort 2 (hazard ratio 1.18; 95% CI 0.4–3.6; *P* = 0.769). Potential confounding effects of other covariates were assessed through multiple bivariate analyses. After adjusting for baseline AST/ALT levels, baseline CD4^+^ count, alcohol use, BMI, HIV risk factors, diabetes, age, sex, and HBsAb positivity, pOBI remained unassociated with the risk of transaminase elevation, with minimal changes in the hazard ratio estimates for both cohorts (Table 2). Notably, pOBI also remained unassociated with the outcome after additional adjustments for HBsAb and the interaction term between HBsAb and HBcAb presence.

**Table 2:** Results from univariate and bi-variate Cox regression analyses exploring the association between occult hepatitis B virus infection and grade ≥1 transaminase elevations after adjustment for possible confounders, in cohort 1 (dual regimens with 3TC, left panel) and cohort 2 (dual regimens without anti-hepatitis B virus drugs, right panel).

	Cohort 1: dual regimens with 3TC			Cohort 2 (*N* = 118): dual regimens without anti-HBV drugs		
Parameter	HR	95% CI	*P*	HR	95% CI	*P*
pOBI unadjusted	0.56	0.20–1.54	0.266	1.18	0.38–3.62	0.769
pOBI (bivariate analysis adjusted for)						
Male gender	0.53	0.19–1.45	0.216	1.16	0.37–3.55	0.798
Age (years)	0.76	0.26–2.21	0.611	1.25	0.38–4.08	0.712
Risk factor for HIV infection	0.60	0.21–1.75	0.350	1.21	0.36–4.05	0.751
HBsAb positive	0.55	0.20–1.59	0.281	1.29	0.40–4.12	0.660
Alcohol	0.56	0.20–1.54	0.262	1.16	0.38–3.58	0.794
BMI	0.57	0.21–1.57	0.274	1.09	0.35–3.40	0.878
Diabetes	0.54	0.19–1.49	0.234	1.34	0.43–4.13	0.602
CD4^+^ T cell at baseline	0.57	0.21–1.56	0.272	1.33	0.43–4.17	0.619
ALT at baseline	0.65	0.23–1.81	0.414	0.67	0.18–2.51	0.549
AST at baseline	0.49	0.18–1.36	0.173	1.09	0.35–3.37	0.879

3TC, lamivudine; ALT, alanine aminotransferase; AST, aspartate aminotransferase; CI, confidence interval; HBsAb, hepatitis B surface antibody; HR, hazard ratio; pOBI, potential occult hepatitis B infection.

### Transaminase dynamics after regimen switch

**Fig. 2:**
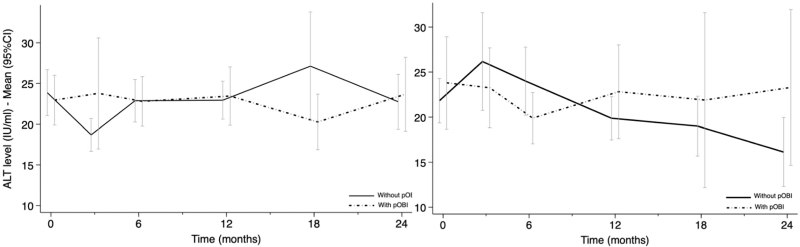
Mean alanine-aminotransferase levels (IU/ml) over 24 months following switch to dual regimens including lamivudine (left panel) and without anti-hepatitis B virus (HBV) drugs (right panel), stratified by the presence of potential occult hepatitis B virus infection.

Figure 2 shows the dynamics of transaminase levels following the switch, illustrating the mean ALT levels in cohort 1 (left panel) and cohort 2 (right panel).

Dashed lines represent those with potential occult HBV infection, while solid lines represent those without. Error bars indicate 95% confidence intervals for mean alanine-aminotransferase levels at each time point. ALT, alanine aminotransferase; CI, confidence interval; HBV, hepatitis B virus; IU/ml, international units per milliliter; pOBI, potential occult hepatitis B infection.

Using mixed-effects modeling revealed no significant difference in ALT levels between patients with and without pOBI in cohort 1 (coefficient 1.64; 95% CI −0.46 to 3.73). Surprisingly, in cohort 2, pOBI was associated with lower mean ALT levels (coefficient −2.57; 95% CI −4.99 to −0.13).

Importantly, there was no evidence of a significant association between time and ALT levels, nor was there a significant interaction between time and pOBI. These findings suggest that the changes in ALT across time did not differ significantly between individuals with and without pOBI.

Using GEE models, we also investigated the likelihood of experiencing grade ≥1 LFTI over time. The results showed that pOBI was not significantly associated with this outcome, with odds ratios of 0.77 (95% CI 0.15–3.99) in cohort 1 and 0.55 (95% CI 0.10–2.56) in cohort 2, thus suggesting that pOBI did not have a meaningful impact on the risk of developing grade ≥1 LFTI in either cohort.

Among the 10 patients with pOBI who experienced increases in liver function tests, nine achieved normalization of their transaminase levels during subsequent follow-up evaluations without any adjustments to their treatment regimen. One patient treated with dolutegravir and lamivudine (cohort 1) showed persistently elevated AST and ALT levels over a 2-year period. Hepatitis B virus reactivation was ruled out through repeated negative HBV DNA tests and HBsAg measurements, and the enzyme elevation was attributed to alcohol use. For five additional patients (two from cohort 1 and three from cohort 2), HBV DNA testing was performed using frozen plasma samples collected around the time of transaminase elevation. None of these patients had detectable levels of HBV DNA. Notably, none of the 10 patients showed HBsAg seroconversion during follow-up.

## Discussion

In our study, we found no evidence that discontinuation of tenofovir – whether alone or alongside lamivudine – is associated with an increased risk of transaminase elevation in patients with pOBI, defined by the presence of anti-HBc antibodies, compared to those without pOBI. Indeed, we observed very few episodes of transaminase elevations after switch to dual therapy. All elevations were mild, with no cases reaching grade 3 or higher. Importantly, we did not observe any occurrences of HBV reactivation in individuals with pOBI and HIV, an event that remains rare and typically limited to cases with specific triggers for reactivation.

While our findings are reassuring, the risk of HBV reactivation cannot be entirely ruled out based on our results or the current literature. Since the early years following the discovery of HIV, cases of seroreversion and reactivation of previously resolved HBV infections have been observed in patients with advanced HIV infection [16,17]. Rare cases of HBV reactivation have been documented even in individuals with well controlled HIV and high CD4^+^ counts, although these are sporadic and often characterized by low viremia without HBsAg reversion [12,18–20].

The most convincing case of HBV reactivation in a nonimmunosuppressed person living with HIV, following with discontinuation of anti-HBV drugs, has been described by Adachi *et al.*[12]. This involved a patient with isolated anti-HBc positivity and undetectable anti-HBs titers who experienced increase in liver function tests and re-appearance of HBsAg few months after switching to long-acting cabotegravir and rilpivirine. Given the patient's high CD4^+^ count and the absence of reported risk factors for reinfection, one plausible explanation is that prior therapy led to apparent, but incomplete, HBsAg clearance, with the chronic HBV infection persisting at a very low level. This unresolved low-grade infection likely facilitated increased viral replication following treatment modification.

Beyond isolated cases, cohort studies have reported virological reactivations in asymptomatic patients, often without associated transaminase elevations or clinical symptoms. For instance, three cases of low-level HBV viremia have been documented in patients with occult HBV infection switching to cabotegravir/rilpivirine, all characterized by extremely low viral loads, no HBsAg reversion, and no transaminase abnormalities [19]. However, lacking longitudinal HBV DNA monitoring or control groups, the clinical significance of such findings remains unclear.

Furthermore, data from a cohort study on anti-HBc-positive/HBsAg-negative individuals with HIV revealed that very low-level HBV viremia, often undetectable with standard assays, can persist even during tenofovir therapy and re-emerge after its discontinuation [20]. Thus, the clinical significance of persistent or fluctuating low-level HBV DNA remains questionable, particularly in the absence of biochemical or clinical correlates.

Rates of HBV reactivation among PWH vary across studies. For instance, an analysis of the Veterans Aging Cohort Study found a low reactivation rate (1.6%) among anti-HBc-positive PWH who switched to HBV-sparing regimens, with significantly higher risk in those with a history of recent prior HBsAg positivity [21]. Similarly, a reactivation rate between 1 and 2% among HBV-exposed individuals, mostly in individuals with HIV virologic failure, has been described in another US cohort. Notably, only half of the cases described were associated with the withdrawal of lamivudine or tenofovir [22]. Other studies reported reactivation rates less than one case/100 patient-years of observation [23]. Taken altogether, these findings suggest that the overall risk of HBV reactivation among people with pOBI and well controlled HIV infection is very low, with clinically significant reactivation being even rarer. This supports the notion that switching to HBV-sparing regimens is not contraindicated in patients without additional risk factors, although careful patient selection or individualized risk assessment could be advisable, particularly in individuals with a history of recent HBV exposure or HBV replication persistence.

It is important to clarify that our findings apply specifically to patients with pOBI and not to inactive HBV carriers or to the risks associated with acute HBV infections in nonimmune individuals. Other studies have analyzed heterogeneous scenarios, including acute infections and reactivations in HBsAg-positive individuals, which are not directly comparable to our context [9]. Similarly, studies reporting unexpectedly high reactivation rates in anti-HBc-positive individuals often lack critical controls, are conducted in high-prevalence settings where reinfections may confound results, or define reactivation solely based on detectable HBV DNA without clear clinical correlates [18]. These methodological differences highlight the need for cautious interpretation when comparing such findings to our results.

The absence of clinically significant transaminase elevations in our study is not unexpected and aligns with the nature of the reported cases of HBV reactivation after discontinuation of HBV-active drugs in immunocompetent patients with pOBI. Unlike the more pronounced reactivation events observed in immunosuppressed individuals, which show the typical pattern of de novo HBV reactivation, usually marked by significant ALT elevations, clinical hepatitis flares, and, in some cases, hepatic decompensation, the cases reported so far appear more consistent with relapses after incomplete viral clearance in individuals who have not achieved a full functional cure.

In our cohort, we did not observe HBV DNA reactivation in patients with elevated transaminase levels, though systematic measurement was not performed in all cases. Although this is a potential limitation of our study, transient, low-level HBV replication events may occur in individuals with pOBI, potentially involving the release of incomplete virions. Highly sensitive assays have detected cryptic serum HBV DNA in up to one-third of anti-HBc-positive/HBsAg-negative patients, suggesting that such low-level replication often persists without clinical significance in the absence of detectable HBsAg or HBV DNA by standard assays [24]. This suggests that routine HBV DNA testing with highly sensitive methods may not be particularly informative, as it could lead to unnecessary concern and the misclassification of HBsAg-negative individuals with normal transaminase levels as early reactivations. In clinical practice, reliance on standard HBV DNA assays is generally appropriate in individual at higher risk of reactivation, and in cases without ALT elevations, repeat testing may help confirm reactivation, particularly if rising levels are observed. Alternatively, in lower risk individuals, a more targeted strategy, reserving HBV DNA testing for those who develop persistent or unexplained transaminase elevations, may be a reasonable and practical approach to identifying true and clinically relevant reactivation events.

Some experts propose limiting HBV reactivation monitoring in individuals with pOBI to those with undetectable HBsAb, given the assumption that HBsAb significantly reduces the risk of reactivation. While our cohort did not show evidence that HBsAb levels modulate transaminase levels or influence the risk of transaminase elevation in individuals with pOBI, the limited number of patients with isolated anti-HBc in our study does not allow definitive conclusions to be drawn regarding this subgroup. Notably, the protective role of HBsAb positivity against HBV reactivation is well documented in immunosuppressed populations. For example, a systematic review demonstrated significantly lower HBV reactivation rates in patients with positive anti-HBs compared to those with negative anti-HBs, across various immunosuppressive settings [25]. Based on these findings, monitoring anti-HBs titers has been suggested as a strategy to predict seroreversions in specific high-risk contexts, such as allogeneic hematopoietic stem cell transplantation [26]. However, among PWH, reactivation cases have been reported even in HBsAb-positive individuals [3,18,22]. Moreover, a notable proportion of individuals with pOBI lack detectable serological markers, with up to 20% reported as serologically negative in previous works [27]. These findings underscore the complexity of assessing HBV reactivation risk in PWH with pOBI and suggest that relying solely on HBsAb may provide an incomplete picture.

Our study has some limitations that should be acknowledged. Its observational design precludes us from fully ruling out residual confounding or selection bias. Furthermore, our cohort generally included individuals with well controlled HIV infection, characterized by virologic suppression and high CD4^+^ cell counts, which may limit the applicability of our findings to PWH who are more severe immunocompromised and potentially at greater risk of HBV reactivation. We also did not evaluate all potential contributors to transaminase elevation, such as metabolic dysfunction-associated steatotic liver disease (MASLD), although we accounted for indirect metabolic markers like diabetes and BMI. In addition, only a small number of participants had isolated anti-HBc antibodies, which limits our ability to draw firm conclusions about this subgroup, where the risk of HBV reactivation may differ. Lastly, the relatively short follow-up period for transaminase elevation may not adequately capture the long-term consequences of diminished control over HBV replication in hepatocytes. Nonetheless, our analyses demonstrated consistency across various assessments, reinforcing the validity of our conclusions. This included evaluating the time to the first transaminase variation, comparing transaminase trends over time between patients with and without pOBI, and analyzing hyper-transaminasemia events using repeated transaminase measurements during follow-up.

In conclusion, our findings provide reassurance that dual regimens without HBV-active agents can be safely used in patients with pOBI and HIV, complementing prior clinical case reports of HBV reactivation, which remain exceedingly rare. Thus, pOBI should not be considered a barrier to switching antiretroviral regimens, as it did not appear to increase the risk of transaminase elevation or alter transaminase levels in this population, although our findings are generalizable in particular to individuals with both anti-HBc and HBsAb positivity, who represented the majority of our cohort. Individual risk assessment remains nonetheless essential, particularly in the presence of additional risk factors. Importantly, excluding individuals with pOBI from clinical trials may bias future data and limit access to innovative therapies for PWH. The absence of significant HBV-related events in our cohort underscores the safety of transitioning to regimens without HBV-active drugs in patients with pOBI and well controlled HIV.

## Acknowledgements

L.M. drafted the manuscript and contributed to data collection and result interpretation. A.S. helped to conceptualize the study and collect the data. A.R., F.S., S.L., A.C., E.C., N.S., I.C.G.C., B.M., and A.C. contributed to data collection. S.M. and A.C. performed laboratory analyses. P.B. supervised the work and contributed to result interpretation. G.L. conceptualized and supervised the study, contributed to data collection, conducted data analysis, interpreted the results and refined the manuscript. All authors reviewed, contributed to, and approved the final version of the manuscript.

### Conflicts of interest

There are no conflicts of interest.
